# Highly Photostable Carbon Dots from Citric Acid for Bioimaging

**DOI:** 10.3390/ma15072395

**Published:** 2022-03-24

**Authors:** Federico Fiori, Hind Moukham, Federico Olia, Davide Piras, Sergio Ledda, Andrea Salis, Luigi Stagi, Luca Malfatti, Plinio Innocenzi

**Affiliations:** 1 Laboratory of Materials Science and Nanotechnology, CR-INSTM, Department of Biomedical Sciences, University of Sassari, Viale San Pietro, 07100 Sassari, Italy; f.fiori1@studenti.uniss.it (F.F.); hind.moukham@gmail.com (H.M.); lstagi@uniss.it (L.S.); luca.malfatti@uniss.it (L.M.); 2 Department of Veterinary Medicine, University of Sassari, Via Vienna 2, 07100 Sassari, Italy; fedeolia92@gmail.com (F.O.); dav.p991@gmail.com (D.P.); giodi@uniss.it (S.L.); 3 Dipartimento di Scienze Chimiche e Geologiche, CSGI, Università di Cagliari, Cittadella Universitaria, SS 554 Bivio Sestu, 09042 Monserrato, CA, Italy; asalis@unica.it

**Keywords:** carbon dots, bioimaging, photoluminescence, cytotoxicity

## Abstract

Bioimaging supported by nanoparticles requires low cost, highly emissive and photostable systems with low cytotoxicity. Carbon dots (C-dots) offer a possible solution, even if controlling their properties is not always straightforward, not to mention their potentially simple synthesis and the fact that they do not exhibit long-term photostability in general. In the present work, we synthesized two C-dots starting from citric acid and tris (hydroxymethyl)-aminomethane (tris) or arginine methyl ester dihydrochloride. Cellular uptake and bioimaging were tested in vitro using murine neuroblastoma and ovine fibroblast cells. The C-dots are highly biocompatible, and after 24 h of incubation with the cells, 100% viability was still observed. Furthermore, the C-dots synthesized using tris have an average dimension of 2 nm, a quantum yield of 37%, high photostability and a zeta potential (ζ) around −12 mV. These properties favor cellular uptake without damaging cells and allow for very effective bioimaging.

## 1. Introduction

Fluorescent carbon dots (C-dots), because of their wide range of emissions and high quantum yields, are opening new possibilities in several biological applications and nanomedicine, especially as markers for bioimaging [1,2,3]. The synthesis of C-dots is relatively simple and allows for the good design of the structure-properties relationship as a function of the applications. However, correlating the origin of the fluorescence with the structure is not a simple task because, notwithstanding the easy processing, the emission can be originated by several sources and has different color centers. Much attention is also required regarding the controlling of the size distribution, composition and the surface functionalization of C-dots. The apparent simplicity of the synthesis of C-dots actually hides several pitfalls; one of these is controlling their intimate structure, another is being able to define a clear correlation between their structure and properties.

The efficiency of carbon dots for bioimaging is provided by a combination of different parameters [4]. It is, in fact, important not only that C-dots are highly fluorescent but that they satisfy several requirements, such as low cytotoxicity, no environmental impact, high quantum yield, and high stability under long exposures to visible and UV light. The emission can be also tuned in different colors, from blue to green and red [5,6].

Bioimaging is used to observe subcellular structures such as organelles that provide essential information about cell metabolism and monitor their responses to external stimuli or therapies in real-time. Up until now, organic dyes have been the first choice; however, their lack of photostability [7], cytotoxicity [8], and low brightness present severe limitations to be overcome. In particular, their low photostability does not allow for the long-term monitoring of cellular functions due to photobleaching because of the damage dealt with the dye structure. Alternative nanoparticles used for bioimaging are semiconductor quantum dots (SQDs) that are characterized by their bright fluorescence, size-tuneable light emission and simultaneous excitation of multiple fluorescence colors [9]. SQDs are more photostable than organic dyes or fluorescent proteins, but their potential cytotoxicity remains a significant drawback. Environmental issues represent another concern because of the heavy metals used in SQD compositions, such as Cd, Pb or Te.

C-dots represent a possible alternative, and they can be applied both for living and apoptotic cells [10] with reduced toxicity. Another intrinsic advantage is their lack of environmental issues and potential low fabrication costs. Their photostability, in particular, is a significant concern because it depends on several structural parameters and the state of the surface of C-dots. In a previous article, we observed that the functionalization of C-dots with 3-aminopropyltriethoxysilane (APTES) increases their photostability. In hydrolytic conditions, the modification of the surface of C-dots by APTES forms a thin passivating layer, which protects the C-dots from emission quenching [11]. In the present work, we focused on obtaining C-dots that could fulfil most in vitro and in vivo bioimaging requirements. In particular, we obtained C-dots that exhibit a remarkable quantum yield and high photostability, which is generally a matter of concern for C-dots. The nanoparticles described in the present work satisfy the main requirements for applications in bioimaging.

## 2. Materials

Citric acid anhydrous (CA, 99.5–100.5%) and tris (hydroxymethyl)-aminomethane (tris, >=99.5%) were purchased from Carlo Erba. L-Arginine methyl ester dihydrochloride (arg, >=98%), were all purchased from Sigma Aldrich. Milli-Q water was used for synthesis and analysis. Dialysis tubing cellulose membrane (molecular weight cut-off = 14,000 Da, Sigma Aldrich) was used for the purification processes.

Ovine adult fibroblast: primary isolation (Department of Veterinary Medicine, University of Sassari, Sassari, Italy); murine C1300 neuroblastoma, was kindly provided from prof. Sergio Gadau (Department of Veterinary Medicine, University of Sassari).

Dulbecco’s modified Eagle’s medium (DMEM) and Ham’s F-12 nutrient mixture (DMEM/F-12), heat-inactivated fetal bovine serum (FBS), glutamine, penicillin/streptomycin, T75 flask, trypsin, falcons, diameter circular coverslips, 60 mm Petri dish, Burker chamber, formaldehyde (4%), thiazolyl blue colorimetric (MTT) and glycerol were all purchased from Sigma Aldrich. All the reagents were employed as received without purification.

### 2.1. Synthesis of Carbon Dots

**Figure 1:**
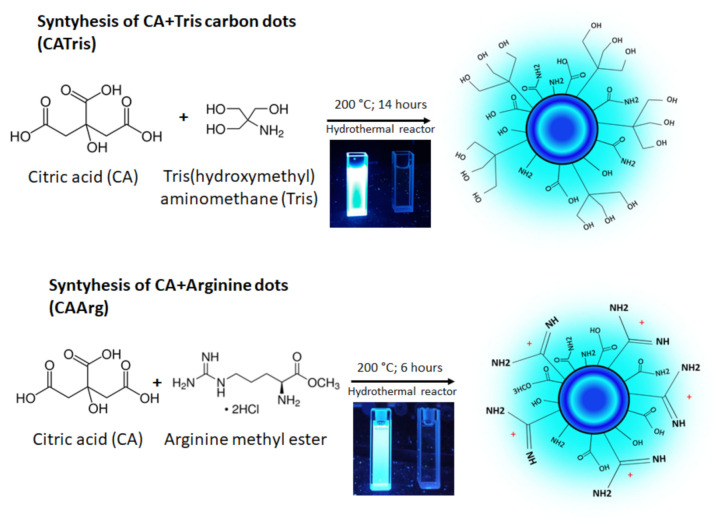
Schematic representation of the synthesis of CATris and CAarg.

C-dots were synthesized from citric acid following two known routes with some modifications: one reacting citric acid with tris (hydroxymethyl) methyl aminomethane (CATris) [12,13,14] and a second one reacting citric acid with arginine methyl ester (CAArg) [15] in a stainless-steel hydrothermal reactor (25 mL volume) with a Teflon reaction vessel. The reactants were sonicated up to complete dissolution in 10 mL of deionized water, transferred in the autoclave, and then placed in a muffle (Ney, Vulcan 3-550) at 200 °C for 14 h for CATris and 6 h for CAArg (Figure 1). After cooling down at 25 °C, the solutions were dialyzed with deionized water as the purifying agent for 24 h to remove small molecular weight compounds and inorganic ions. Carbon dots were obtained at the end of the process. Afterward, the pure solution was freeze-dried with a Lio 5P device and the resultant powders were stored in the dark.

### 2.2. Characterization Techniques

UV-Vis spectra of pure carbon dots were measured in absorbance mode from 200 to 600 nm, with a bandwidth of 1.5 nm, by a Nicolet Evolution 300 spectrophotometer. The C-dots were diluted with deionized water and the data were collected using the same quartz test cuvette.

FTIR spectra were collected in ATR (attenuated total reflection) mode by a Bruker Vertex 70 spectrometer in the range of 4000–400 cm^−1^, with a resolution of 4 cm^−1^ and 32 scans using dried powders of the samples.

Fluorescence spectroscopy measurements were performed using a Horiba Jovin Yvon Fluoromax-3; data were collected in an excitation/emission range of 200–600 nm. Measurement settings, such as slit and increment, were fixed in the different samples to improve the quality of the resultant data in the graphics, with previous 2D studies performed by the same spectrofluorometer.

Photoluminescence quantum yield (QY%) was measured using the quanta-φ (HORIBA) integrating sphere, attached to the “NanoLog” Horiba Jobin Yvon spectrofluorometer. Measurements were performed on equal amounts of water and aqueous solutions of the samples in the same fused silica cuvette. Samples were excited with a wavelength corresponding to their maximum excitation peak and emissions were typically recorded from 200 to 600 nm. The absolute external quantum yield (QY) was calculated using the following equations:

QY(%) = P_1_/P_0_ × 100 (1)

where P_1_ and P_0_ are the integrated intensities of the emitted and incident photons, respectively.

Photostability measurements were taken using a UV-lamp (Spectroline ENF-280C/FE, 230 Volts, 50 HZ, 0.17 AMPS) with a 365 nm excitation light (one 8-watt BL, BLE8T365). The samples (aqueous solutions of the pure carbon dots) were kept in front of the UV light, in a dark room, for several hours. The relative fluorescence intensities acquired at different times were compared. The fluorescence intensities were collected in 2D mode, keeping the excitation wavelength constant to the maximum absorption peak of the samples. Furthermore, the measurement settings of the spectrofluorometer, such as the slit and increment, the cuvette, and the distance between the samples and UV-lamp were kept constant for all the measurements. Data concerning the fluorescence intensities were then reported in a percentage scale.

The zeta potential (ζ) and hydrodynamic diameter (size) of C-dots in solution were measured, at least in triplicate, by using a Zetasizer Nano ZSP instrument (Malvern Instruments) in the backscatter configuration (θ = 173°; laser wavelength = 633 nm). The scattering cell temperature was kept at 298 K, and the data were analyzed using the Zetasizer software 7.03. The samples were prepared by dissolving dried C-dots in Milli-Q water (1 mg mL^−^^1^). The samples were left under rotation for one hour at 25 °C before analysis.

### 2.3. Cell Culture

Primary fibroblast cultures were obtained from a biopsy of tissue collected from adult sheep (ear). The biopsies were washed in 70% ethanol, then two washes were performed in PBS followed by two washes in DMEM/F-12. Subsequently, the biopsies were placed inside a 90 mm glass dish and were sliced into 1 mm fragments using a scalpel. The fragments were carefully moved into a 60 mm tissue culture petri dish, 1 mL of fetal bovine serum (FBS) was added and the dish was placed in an incubator at 37 and 5% CO_2_. After 24 h, the serum was removed and replaced with DMEM/F-12. After 5 days, the tissue fragments were removed, and the cells migrated from the tissues were allowed to grow until the 80% confluence was reached. Subsequently, the cells were trypsinized and passed into 75 cm^2^ flasks, in which they were kept until the sixth cell culture passage. Subsequently, cells were trypsinized and suspended in DMEM-F12 supplemented with 10% DMSO, to be cryopreserved and stored in liquid nitrogen (LN2).

For the bioimaging experiments, thawed ovine fibroblasts and murine C1300 neuroblastoma cells in a T75 flask in DMEM/F-12 were supplemented with 10% FBS, 2 mM glutamine and 100 U/mL penicillin/streptomycin. Cells were trypsinized (0.25% trypsin) and loaded in a 15 mL Falcon tube to be centrifuged for 6 min at 900 rpm at a temperature of 37 °C. Subsequently, the supernatant was removed, and the cells were resuspended in 8 mL of DMEM F12 medium. At the same time, a cell growth system was set up consisting of cleaned 15 mm diameter circular coverslips placed inside a 60 mm Petri dish. Cell counting was then performed using a Burker chamber and approximately 3 × 10^4^ cells were deposited in the 60 mm Petri dish containing the coverslip. After that, the cells were incubated for 24 h in a humidified tissue culture incubator at 37 °C in an air atmosphere with 5% CO_2_.

### 2.4. Cell Imaging

The cell imaging experiment was carried out with a confocal microscope (Leica), both in bright-field and fluorescence, with λ_ex_ = 400 nm and a size of 385.5 µm. Carbon dots were diluted in DMEM-F12 to a concentration of 1 and 3 mg mL^−1^, and then 1 mL of the above solution was added to each Petri dish. After 4 and 24 h of incubation, the cells adhered to coverslips were washed twice thoroughly with PBS. Paraformaldehyde (4%) was added to fix the cells for 10 min at room temperature. After one drop of mounting medium (containing 1 mL of glycerol diluted with 9 mL of water) was added to the microscope slides, the coverslip was placed onto the slides, avoiding air bubbles. The edges of the coverslip were sealed with nail polish and the specimens were stored in the dark at 4 °C. Cells without carbon dots were used as the control of the experiment.

### 2.5. Cytotoxicity

The cytotoxicity of the two types of carbon dots was quantitatively evaluated by thiazolyl blue colorimetric (MTT) assay. Firstly, cells were seeded in a 7000 cells/well 96-well plate. Then, they were separately treated with different concentrations (0/0.2/0.5/1/2 mg mL^−1^) of CATris and CAArg for 24 h. MTT stock solution was prepared by dissolving 10 ul MTT in PBS in a 5 mg mL^−1^ concentration. Then, the stock solution was diluted with a fresh culture medium to reach a final concentration of 0.5 mg mL^−1^. After the culture medium was removed from the wells, a volume of 200 µL/well (incubation solution) was added to each well and they were incubated for another 2 h at 37 °C. Next, after removing the incubation solution, 100 μL of dimethyl sulfoxide (DMSO) was added to each well and mixed by pipetting up and down to permit evidence of crystal formation. The optical density at 490 nm was recorded on a microplate reader.

## 3. Results and Discussion

The synthesis of C-dots was designed to obtain highly fluorescent materials with an emission that is stable over time. The formation and the surface passivation of the C-dots occurred simultaneously, resulting in intrinsic fluorescence emission. Citric acid represented a common source to form a carbonaceous core of the dots, while tris (hydroxymethyl) aminomethane was chosen for the possible formation of a dendritic structure of the carbon dot surface and has been reported to be the source of its great stability [13]. Arginine, instead, has a guanidinium group that is characterized by stronger interactions with the cell membranes, thus providing an efficient uptake [16,17].

**Figure 2:**
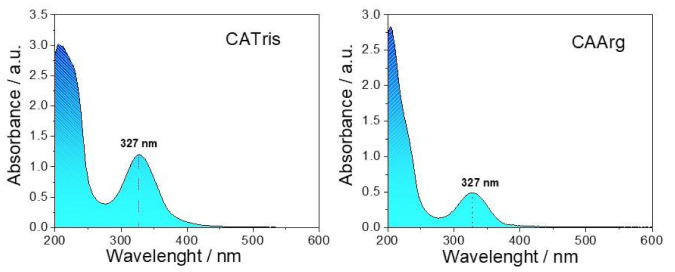
UV-Vis absorption spectra of CATris (**left**) and CAArg (**right**) in aqueous solution.

Figure 2 shows the UV-Vis absorption spectra of CATris (Figure 2 left) and CAArg (Figure 2 right) in the 200–600 nm range. Both C-dots species display two main absorption bands in the UV interval. The most intense absorption at around 205 nm is assigned to π-π*^4^ transitions, whilst the band at 327 nm is attributed to *n*–π*. These particular features are characteristic of CA [18,19] and tris-derived C-dots, which have been proved to have a carbon core and amorphous *sp*^3^ whose extension depends on the synthesis conditions [13,14]. The comparison of the UV-Vis spectra confirms that arginine can also undergo similar interactions to tris with citric acid under the same reaction conditions. In particular, arginine provides both carboxyl and amino groups that form the amide linkage at high temperatures [20] and, in turn, causes the appearance of low energy UV absorption bands in nitrogen rich C-dots.

**Figure 3:**
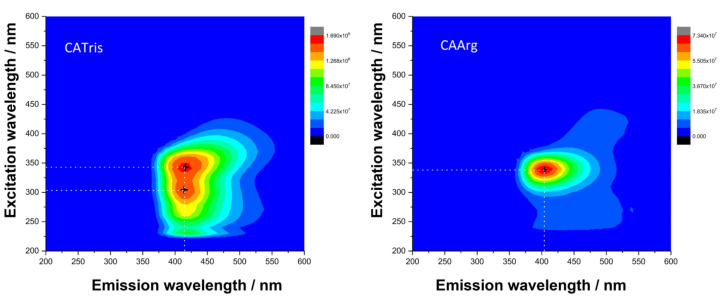
3D fluorescence spectra (excitation (y-scale), emission (x-scale) and intensity (false colour scale)) of CATris (**left**) and CAArg (**right**).

Figure 3 shows the 3D fluorescence spectra (excitation (y-scale), emission (x-scale) and intensity (false colour scale)) of CATris and CAArg. Under excitation at 343 nm, CATris exhibits an emission peaking at 415 nm in the blue range. This emission can also be efficiently excited at 300 nm. CAArg dots show similar blue emissions, with a maximum of 404 nm. Unlike the CATris dots, arginine provides a more symmetric band around the excitation maximum, at around 340 nm. In general, the photoluminescence and quantum yield (QY) of C-dots can be attributed to different mechanisms and depend on the reagents and the methods used to prepare the nanoparticles. Both the CAArg and CATris dots, however, were synthesized by a hydrothermal synthesis that involves the carbonization of N-source precursors. Thermally synthesized CATris dots show a molecular-like fluorescence, which has been already correlated to the formation of polymeric species bearing amide and ester bonds [12]. Correspondingly, the formation of polymeric species assisted by arginine is the origin of the fluorescence in CAArg [21]. It is therefore reasonable to assume that small differences in the kinetics of formation of these species affect the photophysical properties of the two systems.

The quantum yield, which represents a crucial parameter for bioimaging, is an absolute 37% for the CATris and an absolute 20% for CAArg. These are among the most efficient C-dots employed for bioimaging reported so far [22]. The QYs of C-dots with similar compositions are listed in Table S1 of the Supplementary Files.

**Figure 4:**
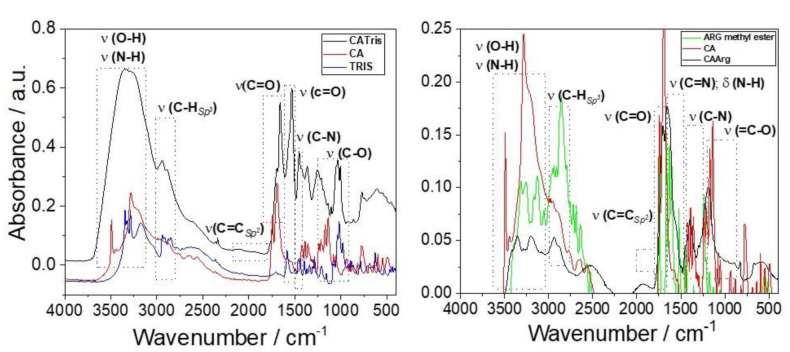
FTIR absorption spectra of CATris (**left**) and CAArg (**right**) samples. The spectra of citric acid, Tris and Arg methyl ester are shown for reference.

Figure 4 shows the FTIR absorption spectra of CATris and CAArg C-dots compared to their reactants. An intense absorption band peaking around 3300 cm^−1^ characterizes the CATris spectrum. The band is attributed to ν_s_(OH) stretching overlapped with the H-bonding band of the amide. The secondary amides formed during polymerization account for this band, agreeing with our previous results [12]. The CATris FTIR spectrum also shows two absorption bands not observed in the CA and tris before the hydrothermal synthesis. The spectrum of CAArg C-dots shows the same vibrational modes; however, they overlap more than in the spectrum of CATris C-dots due to the cationic amine groups of arginine [15]. Below 1500 cm^−1^, the spectra of both CATris and CAArg are characterized by the stretching of C-N, around 1400 cm^−1^, and O=C-O, around 1200 cm^−1^.

**Figure 5:**
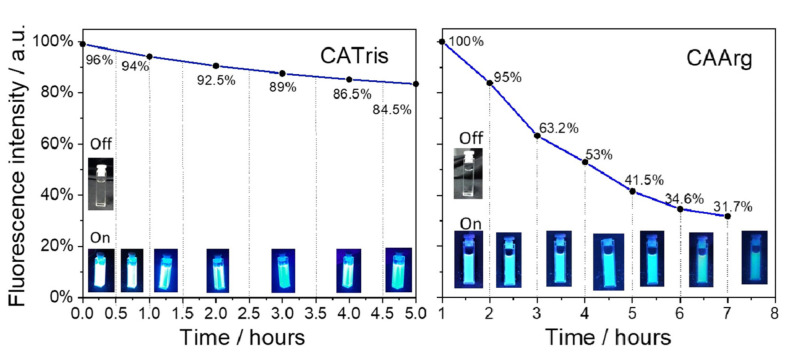
Photostability under UV exposure for CATris (**left**) and CAArg (**right**) carbon dots. The line in CATris represents the exponential decay fit and in CAArg it is a guide for eyes.

The photostability of luminescent nanomaterials is one of the most important factors in evaluating their possible range of applications. In addition, to assess the efficacy of C-dots as potential fluorescent probes for cell and tissue imaging, it is essential to evaluate their photostability. In accordance with the literature, photostability has been evaluated by considering the fluorescence emission intensity during lengthy continuous excitation^2^. In the present study, CATris and CAArg were exposed to UV light for different periods of time to test their photostability (Figure 5). The fluorescence intensity of CATris decreased only slightly, and the C-dots still retained 84.5% of the initial emission intensity even after 5 h of irradiation. CAArg, instead, shows a lower photostability and the fluorescence intensity sensitively decreases over time; after 6 h, the emission intensity decreased to 34.6% of the initial intensity. Beyond these differences, they both are stable enough to be used for bioimaging experiments. The experimental data points of CATris C-dots can be fitted using a nonlinear curve (exponential decay):

y = A_1_exp(−x/t_1_) + y_0_ (2)

which gives A_1_ = 0.226, t_1_ = 4.097, y_0_ = 0.770 with a reduced χ^2^ = 4.22 × 10^−5^.

The high photostability exhibited by CATris is very important because, despite the versatility of organic dyes in the staining of living and fixed cells, their susceptibility to photobleaching represents an inherent problem that limits the applications of organic dyes in cell imaging for long durations. This is in accordance with previous works showing that the chemical composition and steric effects deriving from surface hydroxyl groups (-OH) and dendron-like structures are responsible for photostability [13].

**Figure 6:**
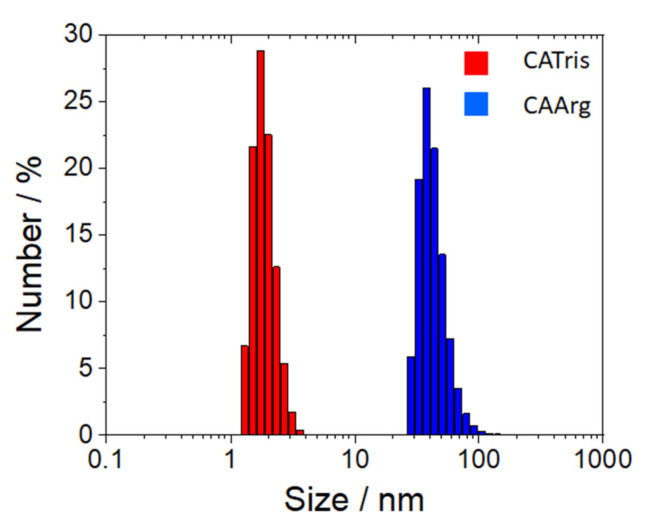
Dynamic light scattering analysis of CATris (red) and CAArg (blue) filtered samples.

The dynamic light scattering analysis of CATris and CAArg (Figure 6) shows that the two C-dots have a sharp dimensional distribution, peaking at around 2 and 40 nm, respectively. The two C-dots, despite having been synthesized and purified using similar protocols, display different dimensions. This effect may be due to the different functional groups of the tris and arginine methyl ester. Under hydrothermal synthesis, the latter could promote the formation of longer polymeric species and larger particles. The internalization of fluorescent C-dots is mainly due to an endocytosis mechanism, including clathrin-mediated endocytosis, caveolae-mediated endocytosis, and clathrin-/caveolae independent endocytosis [23]. Caveolae invaginations of the membranes, involved in the uptake of exogenous materials, show a diameter of 60–80 nm with a 10–50 nm diameter neck [24], which is larger than the average size of our C-dots.

The surface charge of the carbon dots is a crucial factor for both extracellular and intracellular processes such as internalization through charged membranes and interactions with organelles such as mitochondria and the nucleus. Many of these components have, in general, an overall negative charge [25]; for example, the average resting membrane potentials are between −40 mV to −80 mV. The zeta potential (ζ) of the C-dots used for bioimaging was measured as: CATris = −12.88 mV; CAArg = −12.74 mV. Both the C-dots are negatively charged with a similar value of ζ, which accounts for their comparable properties in terms of endocytosis and cytotoxicity.

### 3.1. In Vitro Cytotoxicity Studies

**Figure 7:**
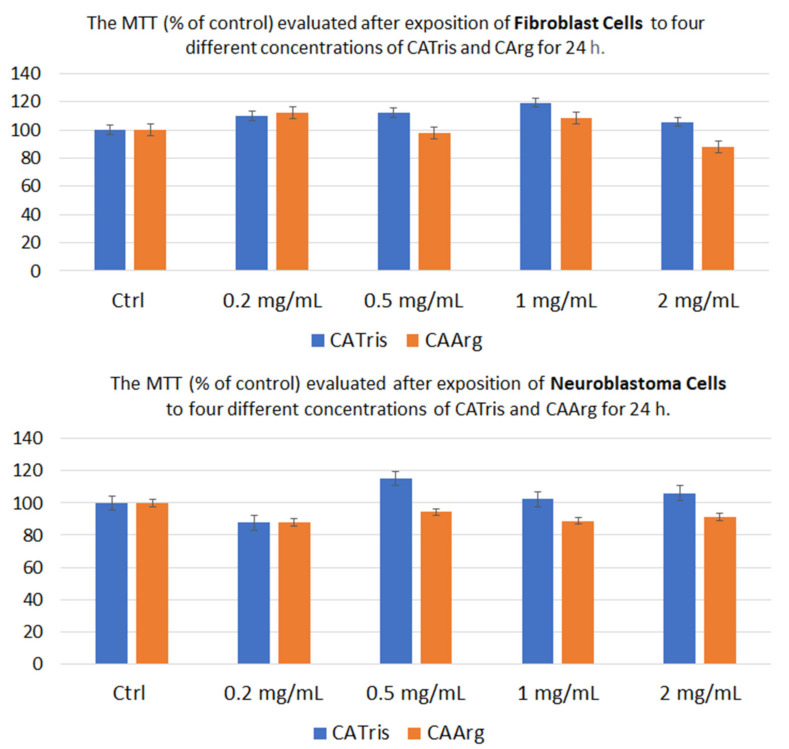
Fibroblast (**top**) and Neuroblastoma cells (**bottom**) from MTT assays with different CATris and CAArg concentrations after 24 h incubation.

The cytotoxicity of carbon dots has attracted considerable attention in recent years and has been investigated by many research groups. C-dots generally have low toxicity in vitro but the related studies are still contradictory. To investigate the potential bio-related applications of the C-dots, we performed in vitro cytotoxicity studies by MTT assay in terms of cell viability, using the two different types of C-dots. Figure 7 shows the relative cell viability of fibroblast cells (Figure 7 top) and neuroblastoma cells (Figure 7 bottom) treated with CATris and CAArg at various concentrations after 24 h of incubation. The experimental data indicate that after 24 h of exposure to C-dots, the proliferation of fibroblast and neuroblastoma cells was statistically similar to the control groups. Even at the highest concentration of 2 mg mL^−1^, more than 85% of the cell MTT (% of control) still remained, implying that CATris and CAARg with different functional groups have a good compatibility and low cytotoxicity, which is due to their intrinsic organic structure and negative zeta potential (ζ) [26].

### 3.2. Bioimaging

Bioimaging represents one of the main fields of application for C-dots, because of their high fluorescence and biocompatibility. In this study, we evaluated the efficiency of CATris and CAArg C-dots using two different cell lines, fibroblast and neuroblastoma cells. The concentration and incubation time are the parameters used to evaluate the cellular uptake.

**Figure 8:**
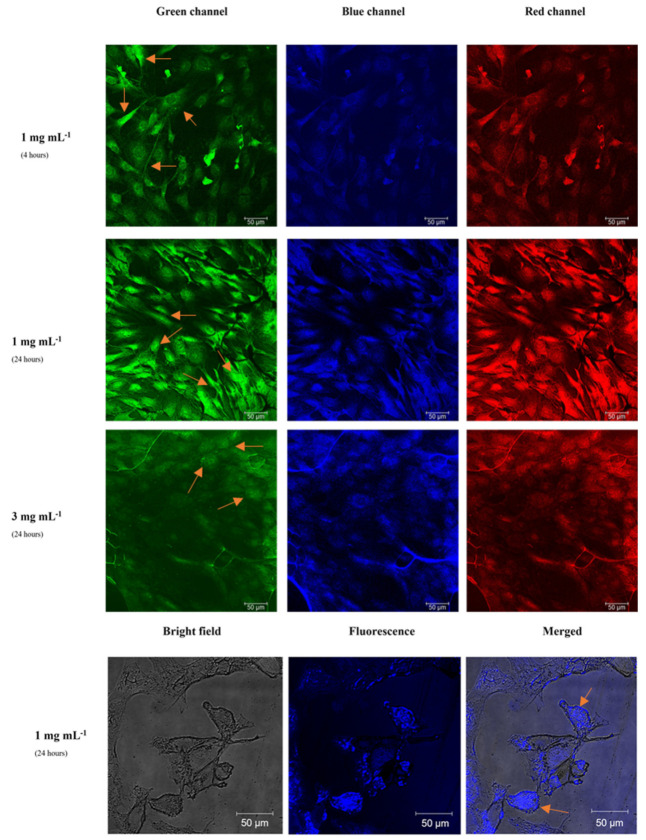
Fluorescence confocal microscopy images of fibroblast cells with internalized CATris, at a concentration of 1 and 3 mg mL^−1^, after 4 and 24 h of incubation under green, blue and red channels. Bottom row: bright field, fluorescence and merged imaging of fibroblast cells with internalized CATris, at a concentration of 1 mg mL^−1^, after 24 h of incubation. (λ_ex_ = 400 nm; size 385.5 µm). Some of the cells showing overall uptake of C-dots have been marked with orange arrows for a better understanding of the images.

Figure 8 shows the images taken by a fluorescence confocal microscope of fibroblast cells with internalized CATris C-dots at a concentration of 1 and 3 mg mL^−1^ after 4 and 24 h of incubation. It is important to note that we did not observe any fluorescence quenching at these concentrations due to aggregation-induced photon reabsorption. This phenomenon, however, has been already observed for C-dots^6^ and likely occurs at higher concentrations. The changes in intensity are due to the different C-dot responses when excited with a λ_ex_ = 400 nm. At this wavelength, in fact, the intensity of the blue emission is weak for both CATris and CAarg (Figure 3), while the intensity of emission at a longer wavelength is higher. The images have been separated according to the green, blue and red channels for a better understanding. The images in the bottom row show the fluorescence and bright-field imaging and the merged imaging (bright-field + fluorescence) of fibroblast cells with internalized CATris, at a concentration of 1 mg mL^−1^, after 24 h of incubation.

The cell imaging experiments demonstrate the successful cell uptake of CATris in fibroblast cell lines (Figure 8); this uptake, as previously demonstrated, does not cause cytotoxic effects. The images obtained by changing the concentration do not show significant differences; therefore, increasing the CATris concentration from 1 to 3 mg mL^−1^ does not result in an effective increase in fluorescence and uptake from the cells. The lower brightness of some images (Figure 8, row 3) is due to the inhomogeneity of the cell culture and the local distribution of carbon dots and cannot be related to the higher concentration of C dots. The concentration of 1 mg mL^−1^ of CATris represents an efficient threshold level for cell labeling. In addition, merged imaging (Figure 8, bottom row) indicates that most of the fluorescence intensity is coming from the membrane and cytoplasm cellular regions, even if some cells with an overall uptake are detected. The ζ-potential plays an important role in the uptake of C-dots. It has been previously reported that positively-charged C-dots are able to reach the cell nucleus [27], whereas neutral C-dots are instead evenly distributed inside the cells and negatively charged C-dots are preferentially located in the cytoplasm [28]. The present results show that the fluorescence originates from the cytoplasm region, which is in accordance with previous findings, thanks to an overall negative charge of our C-dots. On the other hand, the ζ-potential value of −12.74 mV for CATris is what is considered a neutral zone (from −10 mV to +10 mV) [29], and this would explain the observation of some cells showing an even distribution of the C-dots. Furthermore, no significant differences were detected after 4 and 24 h of incubation time (Figure 8, first and second row); hence, we can report that 4 h of incubation is sufficient for cell labeling with CATris. Overall, the images show that the C-dots can easily cross cell membranes and enter into cells within 4 h, resulting in even distribution and the strong illumination of the elongated fibroblasts (Figure 8).

**Figure 9:**
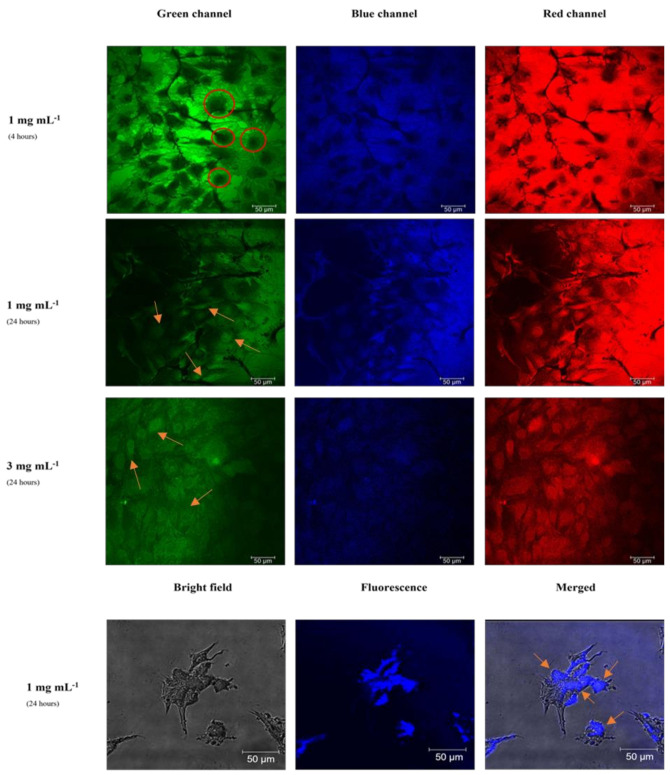
Fluorescence confocal microscopy images of fibroblast cells with internalized CAArg at a concentration of 1 and 3 mg mL^−1^ after 4 and 24 h of incubation under green, blue, and red channels. Few black spots as references are indicated with red circles. Bottom row: Bright field, fluorescence and merged imaging of fibroblasts cells with internalized CAArg, at a concentration of 1 mg mL^−1^, after 24 h of incubation (λ_ex_ = 400 nm; size 385.5 µm). Some of the cells showing an overall C-dots uptake are marked with orange arrows for a better understanding of the images.

The bioimaging experiment was also performed using CAArg C-dots (Figure 9) in conditions identical to those employed for CATris. The cell uptake of CAArg was also successfully detected in fibroblast cell lines but showed a particular behavior compared to CATris. Indeed, no significant differences were detected between the two concentrations, but the incubation time affected the cell uptake.

The obtained images (Figure 9, first and second row) show that CDAArg was gradually taken up by fibroblasts cells from 4 to 24 h. After 4 h, the uptake was observed only in the membrane and cytoplasm. In fact, we can detect several black spots (Figure 9, first row), which indicates a non-even distribution inside the cells. These black spots could be assigned to the nuclei. However, after 24 h, the black spots were fulfilled (Figure 9, second and third row) and uptake was also observed in the nucleus. The uptake was confirmed, in addition, by the merged imaging (Figure 9, bottom row). Therefore, the incubation time is a key factor in the cell uptake of CAArg into the nucleus, maintaining easy access to the cell membranes and cytoplasm. The even distribution of CAarg is in accordance with previous studies [28,29], as CAArg has a ζ-potential of −12.88 mV, similar to CATRis, while the slower distribution can be attributed to the different dimensions and surface species.

**Figure 10:**
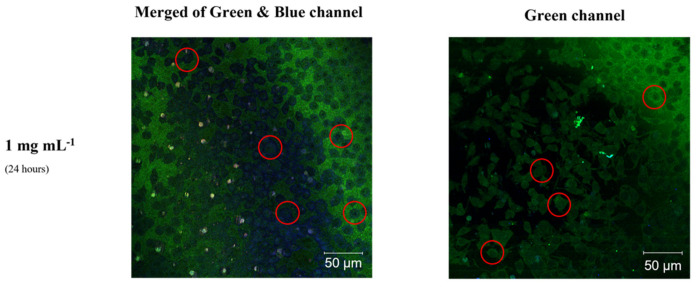
Fluorescence confocal microscopy images of neuroblastoma cells with internalized CATris (**left**) and CAArg (**right**), at a concentration of 1 mg mL^−1^, after 24 h of incubation under merged of green and blue channels (CATris) and green channel (CAArg). (λ_ex_ = 400 nm; size f85.5 µm). Some of the cells showing an overall uptake of the C-dots are marked with red circles for a better understanding of the images.

To comprehensively evaluate the synthesized behavior of the C-dots, CATris and CAArg were tested in tumorous cells, and an overall efficient uptake was observed in the neuroblastoma cell line (Figure 10).

Merged green and blue channels were used (Figure 10, left) to evidence the emission of CATris from that the background, while a green channel only was enough to point out the emission from CAarg (Figure 10, right). The cell uptake of both carbon dots tested, CATris and CAArg, at a concentration of 1 mg mL^−1^, was successfully detected in neuroblastoma cell lines. It was demonstrated that after 24 h of incubation, CAtris and CAarg efficiently distributed into tumorous cells, highlighting the spherical morphological characteristics of the neuroblastoma cells. The uptake was homogeneous, as expected by the value of the ζ-potential [28,29]. The C-dots’ emission was in the blue and the green range for CATris (Figure 10, left) and CAarg (Figure 10, right), respectively.

This result is a good indication of the potential dual-use of C-dots in bioimaging and drug delivery for cancer diagnosis and therapy. CAArg, in particular, can be usefully applied for efficient gene delivery because of its guanidinium positive local charges as a ligand factor for the negative charges of the gene’s phosphate groups. Some studies have demonstrated the highly efficient delivery of genetic materials to adenokarcinom cells by arginine-C-dot carriers [30]. Targeting the nucleus with C-dots for drug delivery is essential, especially for cancer treatment. C-dots have been found to be an efficient agent for nucleus labeling and a nanocarrier for nucleus-targeted therapy with or without the post-modification of nuclear-targeting ligands [31]. Our results are in accordance with such findings, showing that C-dots are mostly localized in the cytoplasm, with uptake in the nucleus after a more lengthy incubation period. Internalization can be also achieved by either targeting specific functionalization or by intrinsic functional groups on the C-dots preserved from their precursors [23].

## 4. Conclusions

The present study investigated the cell uptake of two types of C-dots synthesized via a facile and green hydrothermal synthesis, using citric acid and either tris (CATris) or arginine (CAArg) as precursors. CATris and CAArg have an absolute quantum yield of 37% and 20%, respectively, and exhibit favorable biocompatibility, an easily modifiable surface, strong blue fluorescence emission and low cytotoxicity. CATris displayed a better optical performance over CAArg and has a higher photostability under UV irradiation, maintaining up to 84.5% of initial emission intensity after exposure for 5 h under UV light. CATris and CAArg show a similar zeta potential, −12.9 and −12.7 mV, respectively, but different dimensions of around 2 and 40 nm. The ζ-potential value around −12 mV enables the C-dots to be attracted to cell membranes efficiently and for them to evenly dislocate inside the cells. The biocompatibility assay showed that 1 mg mL^−1^ of C-dots can be safely used in biomedical imaging, with an effective illumination of the cells. Furthermore, even at higher concentrations, such as 3 mg mL^−1^, no visible mortality and apoptosis or necrosis resulted from the cytotoxicity MTT-based assay. CATris and CAArg are mainly localized in the cytoplasm, with some nanoparticles detected in the nucleus. The high photothermal stability, the straightforward synthesis and the absence of detectable cytotoxicity at the experimental concentrations make this class of C-dots a good candidate for bioimaging.

## Data Availability

Data are available on request to the corresponding author.

## Supplementary Materials

The following supporting information can be downloaded at https://www.mdpi.com/article/10.3390/ma15072395/s1, Table S1. Quantum Yields of C-dots with a composition similar to CATris and CAArg.
