# Supplementary material for: Highly Photostable Carbon Dots from Citric Acid for Bioimaging
Source: Materials (Basel). 2022 Mar 24;15(7):2395. doi: 10.3390/ma15072395 (PMC9000082; doi:10.3390/ma15072395)
Supplement: Supplementary file 1 [file materials-15-02395-s001.zip › materials-1571740-supplementary.pdf]

materials

MDPI

Supplementary Files

Highly photostable carbon dots from citric acid for bioimaging

Table S1. Quantum Yield of C-dots with composition similar to CATris and CAArg.

Precursors	QY	Ref.
**Citric Acid + Cysteine**	10.73 % (absolute)	[6]
**Tris (hydroxymethyl)- aminomethane + Citric Acid**	84% (relative)	[12]
**Tris (hydroxymethyl)- aminomethane + Citric Acid**	75% (absolute)	[13]
**Arginine + Citric Acid**	29% (relative)	[22]
**Lysine + Citric Acid**	75% (relative)	[22]
**Histidine + Citric Acid**	23% (relative)	[22]
**Cysteine + Citric Acid**	71% (relative)	[22]
**Methionine + Citric Acid**	12.9% (relative)	[22]
